# Comparative Analysis of Deep Neural Networks for Automated Ulcerative Colitis Severity Assessment

**DOI:** 10.3390/bioengineering12040413

**Published:** 2025-04-13

**Authors:** Andreas Vezakis, Ioannis Vezakis, Ourania Petropoulou, Stavros T. Miloulis, Athanasios Anastasiou, Ioannis Kakkos, George K. Matsopoulos

**Affiliations:** 1 Biomedical Engineering Laboratory, School of Electrical & Computer Engineering, National Technical University of Athens, 15773 Athens, Greece; avezakis@biomed.ntua.gr (A.V.); ivezakis@biomed.ntua.gr (I.V.); rpetro@biomed.ntua.gr (O.P.); smiloulis@biomed.ntua.gr (S.T.M.); aanastasiou@biomed.ntua.gr (A.A.); ikakkos@biomed.ntua.gr (I.K.); 2 Department of Biomedical Engineering, University of West Attica, 12243 Athens, Greece

**Keywords:** ulcerative colitis, Mayo endoscopic score, disease severity assessment, endoscopy, deep learning, neural networks, classification

## Abstract

Background: Ulcerative colitis (UC) is a chronic inflammatory bowel disease characterized by continuous inflammation of the colon and rectum. Accurate disease assessment is essential for effective treatment, with endoscopic evaluation, particularly the Mayo Endoscopic Score (MES), serving as a key diagnostic tool. However, MES measurement can be subjective and inconsistent, leading to variability in treatment decisions. Deep learning approaches have shown promise in providing more objective and standardized assessments of UC severity. Methods: This study utilized publicly available endoscopic images of UC patients to analyze and compare the performance of state-of-the-art deep neural networks for automated MES classification. Several state-of-the-art architectures were tested to determine the most effective model for grading disease severity. The F1 score, accuracy, recall, and precision were calculated for all models, and statistical analysis was conducted to verify statistically significant differences between the networks. Results: VGG19 was found to be the best-performing network, achieving a QWK score of 0.876 and a macro-averaged F1 score of 0.7528 across all classes. However, the performance differences among the top-performing models were very small suggesting that selection should depend on specific deployment requirements. Conclusions: This study demonstrates that multiple state-of-the-art deep neural network architectures could automate UC severity classification. Simpler architectures were found to achieve competitive results with larger models, challenging the assumption that larger networks necessarily provide better clinical outcomes.

## Simple Summary

Deep learning approaches are revolutionizing medical image analysis, offering potential solutions for standardizing disease assessment in gastroenterology. In ulcerative colitis (UC), a chronic inflammatory bowel disease, accurate severity assessment is crucial for treatment decisions. The Mayo Endoscopic Score (MES) is the current gold standard for evaluating UC severity, but its subjective nature can lead to inconsistent scoring between observers. This study explores various deep learning architectures to develop an automated, objective system for MES classification. By comparing multiple state-of-the-art neural networks, we identify the most effective approach for standardizing UC severity assessment, potentially improving treatment decisions and patient outcomes.

## 1. Introduction

Ulcerative colitis (UC) is a chronic, idiopathic inflammatory bowel disease (IBD) characterized by persistent inflammation of the colonic mucosa. This inflammation primarily affects the rectum and may extend proximally in a continuous manner to involve the entire colon [1]. The underlying etiology of UC remains unclear, but it is thought to result from a complex interplay of genetic, environmental, immune, and microbial factors. The prevalence of UC varies globally, with higher incidence rates observed in Western countries. However, emerging epidemiological data indicate a rising trend, suggesting a possible influence of lifestyle and environmental changes [2]. UC significantly impacts patients’ quality of life, leading to symptoms such as diarrhea, rectal bleeding, abdominal pain, urgency, and fatigue [3]. Additionally, the chronic nature of the disease can contribute to emotional distress, anxiety, and reduced social functioning [4,5]. It is also, a remitting and relapsing disorder, characterized by periods of symptomatic exacerbations followed by phases of clinical remission [6]. Due to this unpredictable disease course, ongoing monitoring is essential for effective disease management. Regular assessment through clinical evaluation, mood-targeted interventions, endoscopic surveillance, and biomarker monitoring helps optimize treatment strategies, prevent complications, and improve long-term outcomes for patients [7,8].

Specifically, endoscopic assessment plays a critical role in the ongoing monitoring of ulcerative colitis (UC), providing direct visualization of mucosal inflammation and aiding in treatment decisions [9]. Mucosal healing, as it is determined by endoscopy, is a key therapeutic target associated with reduced risk of disease progression, complications, and colectomy. The Mayo Endoscopic Score (MES) is the gold standard for evaluating disease severity in UC. This scoring system assesses mucosal inflammation through key visual indicators: erythema (redness of the mucosa), vascular pattern (visibility of blood vessels beneath the mucosa), friability (tendency of tissue to bleed when touched), and the presence of erosions or ulcers [10]. Despite its widespread use, current endoscopic assessment methods present several challenges, particularly related to inter- and intra-observer variability with the level of experience [11]. The subjective nature of endoscopic scoring can lead to discrepancies in disease severity grading between different endoscopists (inter-observer variability) and even between repeated evaluations by the same endoscopist (intra-observer variability). Such inconsistencies can impact clinical decision-making, influencing treatment choices and disease monitoring strategies [12]. Additionally, endoscopic procedures are invasive, costly, and may not always be feasible for frequent monitoring. The MES is a four-tiered grading system (0 to 3) based on mucosal appearance during endoscopy, as follows:

- 0: Normal or inactive disease.
- 1: Mild disease (erythema, decreased vascular pattern, mild friability).
- 2: Moderate disease (marked erythema, absent vascular pattern, friability, erosions).
- 3: Severe disease (spontaneous bleeding, deep ulcers).

Given these challenges, efforts are being made to develop more objective and reproducible assessment tools, including artificial intelligence-assisted endoscopic evaluation and biomarker-based disease monitoring, to complement conventional endoscopic scoring systems [13,14,15,16,17].

To address these challenges, computer-aided diagnosis (CAD) using advanced artificial intelligence (AI) algorithms has emerged as a promising solution [17]. AI-based tools, particularly those utilizing deep learning models, have the potential to enhance the accuracy and consistency of endoscopic assessments. These algorithms can automatically analyze endoscopic images and videos, offering real-time, objective, and reproducible grading of mucosal inflammation. Such systems could help mitigate the issues of inter- and intra-observer variability, reduce human error, and provide consistent disease monitoring.

Recent successes in deep learning for medical imaging have demonstrated its capability to outperform traditional methods in various domains [18,19]. For example, deep learning has shown remarkable performance in detecting and classifying pathologies in radiology and dermatology, where algorithms can identify patterns that are often difficult for human observers to detect [20,21]. Furthermore, in the realm of gastrointestinal (GI) conditions, AI-driven techniques have already been successfully applied to gastroscopy and colonoscopy [22]. Studies have shown that deep learning models can accurately detect colorectal cancer and polyps [23], as well as assist in evaluating conditions such as Crohn’s disease and esophageal cancer [24]. These advancements suggest that AI may serve an important role in enhancing the diagnostic and monitoring capabilities for UC, ensuring more accurate, efficient, and consistent clinical care.

In the present study, several state-of-the-art deep learning models were trained for automated ulcerative colitis (UC) severity classification using endoscopic images. The key focus involves comparing different convolutional neural network (CNN) architectures to determine their effectiveness in accurately assessing disease severity while reducing the subjectivity of conventional scoring systems like the Mayo Endoscopic Score (MES).

## 2. Materials and Methods

### 2.1. Dataset

The Labeled Images for Ulcerative Colitis (LIMUC) dataset, which is publicly available, was utilized for this study [25]. For this dataset, a total of 19,537 endoscopic images were collected from 1043 colonoscopy procedures performed on 564 UC patients at Marmara University Institute of Gastroenterology between December 2011 and July 2019. All images were acquired using a Pentax EPK-i video processor and Pentax EC-380LKp video colonoscope (Pentax, Tokyo, Japan) and standardized to a resolution of 352 × 288 pixels during database storage. The images were captured at different time points during the colonoscopy procedures, ensuring no spatial relationship among images from the same patient and thus increasing dataset heterogeneity.

Images unsuitable for evaluation due to debris, inadequate bowel preparation, artifacts, retroflexion, or poor image quality were excluded from the study. All patient information, software outputs, and temporal data were masked to prevent bias. Two experienced gastroenterologists independently reviewed and classified all images according to the Mayo Endoscopic Score (MES). The inter-reader reliability for MES labeling was measured with quadratic weighted kappa and achieved a score of 0.781. The initial diagnoses from the gastroenterologist who performed the colonoscopy were not used in the labeling of this dataset.

For images with inconsistent labels between the two reviewers (7652 images), a third independent reviewer, blinded to previous classifications, provided an additional assessment. Final scores for these cases were determined using majority voting. Images that received different labels from all three reviewers were excluded from the study.

After applying all exclusion criteria, including 8060 images deemed unsuitable for MES evaluation and 201 images with complete reviewer disagreement, the final dataset consisted of 11,276 images with the following distribution:

- MES 0: 6105 (54.14%).
- MES 1: 3052 (27.07%).
- MES 2: 1254 (11.12%).
- MES 3: 865 (7.67%).

**Figure 1:**
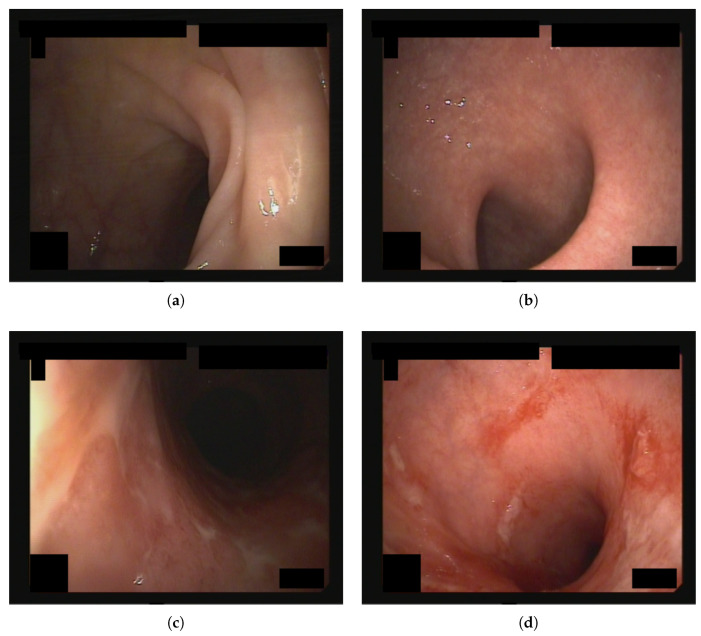
Endoscopic images from the dataset used in this study, each assigned an MES score of (**a**) 0, (**b**) 1, (**c**) 2, and (**d**) 3.

Figure 1 depicts a sample image for each MES evaluation.

### 2.2. Experimental Setup

In this study, the performance of several state-of-the-art deep learning models was evaluated on the LIMUC dataset. The selected models were DenseNet [26], EfficientNet [27], MobileNetV2 [28], ResNet [29], VGG [30], and Vision Transformer (ViT) [31], all of which have demonstrated state-of-the-art results on the ImageNet classification challenge [32]. Transfer learning [33] was utilized by initializing all models with weights pre-trained on ImageNet. To accommodate the LIMUC classification task, the final classification layer of each network was modified by replacing the original 1000-class ImageNet output layer with a layer containing four neurons, one for each MES score.

Neural network scaling affects both capacity and generalization performance [27]. Models with more parameters are usually better at handling complex tasks, but they may also overfit their data, particularly if the datasets are small. For this reason, network architectures come in several variants, which utilize the same base architecture but increase the number of parameters. However, it is not always clear which variant will be the most performant. For this reason, this study evaluates several variants of each model family. The detailed list of the network variants and their total parameters are shown in Table 1.

The dataset was split into training and test sets using an 80/20 ratio. To ensure a fair comparison, all models were trained and evaluated on the same dataset partitions. For training, the Adam optimizer [34] was used with a learning rate of $3\times 10^{-4}$. The training process was conducted on an NVIDIA RTX 4090 (NVIDIA Corporation, Santa Clara, CA, USA) graphics card with 24 GB of VRAM for 300 epochs, utilizing a fixed input image size of (224, 224) pixels and a batch size of 64. However, for EfficientNetB6 and EfficientNetB7, the batch size was reduced to 32 because of insufficient memory. Generally, batch size has been shown to influence the generalization performance of neural networks. Smaller batch sizes lead to noisier gradient updates, which can help escape local minima during training, but may also slow down convergence. Conversely, larger batch sizes typically lead to faster convergence and exhibit a stabler loss trajectory, but can also result in poorer generalization [35]. In this study, all models reached convergence regardless of batch size, suggesting that in this particular setup, this small batch size variation did not substantially affect the final results.

Due to class imbalance in the dataset, cross-entropy loss [36] with class weighting was employed to ensure that underrepresented classes received higher importance during training. Cross-entropy loss, commonly used for classification tasks, measures the divergence between the predicted probability distribution and the true class labels. It is defined as follows:

(1) $$L=-\sum_{i=1}^{N}y_{i}\log\left({\hat{y}}_{i}\right)$$

where $y_{i}$ is the true label (1 for the correct class, 0 otherwise), and ${\hat{y}}_{i}$ is the predicted probability for that class. This loss function penalizes incorrect predictions more severely when the confidence in the wrong class is high, encouraging the model to output probabilities that align closely with the actual distribution of labels.

However, in imbalanced datasets, standard cross-entropy loss can lead to biased learning, where the model favors majority classes and struggles to recognize minority classes effectively [37]. To counteract this, class weighting is commonly applied, where the weight assigned to each class is computed as follows:

(2) $$w_{c}=\frac{N}{K\cdot n_{c}}$$

where *N* is the total number of samples, *K* is the total number of classes, and $n_{c}$ is the number of samples in class *c*. This ensures that classes with fewer samples receive higher weights, thereby balancing the contribution of each class to the loss function.

Further to image resizing, each RGB channel of the inputs was independently normalized by subtracting the mean and dividing by the standard deviation, as computed on the entire training set. The mean was determined as [0.4143, 0.2877, 0.2184] and the standard deviation as [0.2986, 0.2210, 0.1784], for the red, green, and blue channels, respectively. In addition, data augmentation techniques were applied during training to increase the diversity of the training set. The augmentations used were as follows:

- Horizontal flipping: The image was flipped horizontally with a 50% probability of introducing left–right variations.
- Vertical flipping: The image was flipped vertically with a 50% probability of adding top–bottom variations.
- Color jittering: The following image color properties were randomly adjusted as follows:
  – Brightness adjusted between 60% and 160% of the original.
  – Contrast adjusted by a factor of 0.2.
  – Saturation adjusted by a factor of 0.1.
  – Hue slightly altered within a range of ±0.01.
- Elastic transformations: The image structure was deformed in a non-linear fashion to mimic realistic distortions while preserving crucial features, using Lanczos4 interpolation for smooth transitions.

All experiments were conducted using Python 3.12 and PyTorch 2.1.2 with CUDA 11.8 for acceleration.

### 2.3. Network Evaluation

To evaluate model performance, four standard classification metrics were used: F1 score, recall, precision, and accuracy. These metrics were computed individually for each class following a one-versus-rest approach, where each class is evaluated independently by transforming the multi-class problem into a binary classification one. In this approach, instances of the target class are considered positive samples, while instances from all remaining classes are collectively treated as negative samples.

The accuracy of a class is defined as follows:

(3) $$acc=\frac{TP+TN}{TP+TN+FP+FN}$$

The precision of a class is defined as follows:

(4) $$P=\frac{TP}{TP+FP}$$

The sensitivity (recall) of a class is given by the following:

(5) $$R=\frac{TP}{TP+FN}$$

where TP (true positive) denotes the correctly predicted positive case, FP (false positive) denotes the incorrectly predicted positive case, and FN (false negative) denotes the actual positive case that was incorrectly classified as negative.

The F1 score is the harmonic mean of precision and recall, formulated as follows:

(6) $$F1=2\frac{P\cdot R}{P+R}$$

This metric balances precision and recall, making it particularly useful for imbalanced datasets where one class might dominate over others.

Furthermore, to facilitate comparison with the results of other studies, the weighted kappa score [38] was calculated for the best-performing model, as determined by the mean value of the F1 score across all classes. The weighted kappa score is a statistical measure used to assess the agreement between two raters while accounting for the degree of disagreement. Unlike simple accuracy, which only considers correct versus incorrect predictions, the weighted kappa score applies a weighting scheme that penalizes larger discrepancies more heavily. Given the ordinal nature of the MES scores, the quadratic weighted kappa (QWK) was employed, which assigns quadratic penalties to differences between predicted and actual ordinal categories. This makes it particularly useful in tasks such as medical diagnosis, grading systems, and other ordered classification problems.

To estimate the uncertainty associated with the weighted kappa score, bootstrapping was employed. Bootstrapping is a resampling technique that involves repeatedly drawing random samples with replacements from the original dataset to create multiple resampled datasets. By computing the weighted kappa score for each resampled dataset, a distribution of scores is obtained, allowing the estimation of confidence intervals. This approach provides insights into the stability and reliability of the model’s performance, ensuring that reported kappa scores are robust to variations in the data.

#### Statistical Comparison

To assess the statistical significance of performance differences between models, McNemar’s tests were conducted [39]. McNemar’s test is a statistical technique specifically designed for analyzing paired nominal data. The test analyzes the disagreements between two classifiers, focusing on cases where one classifier is correct while the other is incorrect. By analyzing these mismatched predictions, McNemar’s test determines whether the performance gap between two classifiers is statistically significant or just random. The test creates a contingency table, *M*, of agreements and disagreements between the classifiers and then calculates a chi-square statistic to assess whether the observed differences in error patterns are meaningful. The test statistic (chi-square $x^{2}$) and the contingency table *M* are defined as follows:

(7) $$x^{2}=\frac{{\left(b-c\right)}^{2}}{{\left(b+c\right)}^{2}}$$

(8) $$M=\left[\begin{array}{cc} a & b\\ c & d \end{array}\right]$$

where *b* is the number of cases where classifier 1 is correct and classifier 2 is incorrect, *c* is the number of cases where classifier 1 is incorrect and classifier 2 is correct, *a* is the number of cases where both classifiers are correct, and *d* is the number of cases where both classifiers are incorrect.

## 3. Results

### 3.1. Network Performance

The performance metrics of all evaluated models are presented in Table 2, Table 3, Table 4 and Table 5. To determine the optimal model, the macro-averaged F1 score was utilized as the primary evaluation metric. This choice was motivated by the class imbalance in the dataset, as the F1 score provides a more balanced representation of model performance by considering both precision and recall.

Based on the macro-average F1 scores across all MES score classes, the top five performing models were VGG19 (0.7528), EfficientNetB1 (0.7521), EfficientNetB6 (0.7493), MobileNetV2 (0.7472), and DenseNet169 (0.7465). VGG19 demonstrated superior performance particularly for MES 1 and 2, although it was slightly outperformed in MES 0 and 3.

For MES 0, DenseNet201, EfficientNetB3, and EfficientNetB6 achieved the highest F1 scores (0.887), while VGG19 demonstrated the best performance for MES 1 (0.702) and MES 2 (0.668). For MES 3, DenseNet169 attained the highest F1 score (0.792). Notably, VGG19 did not achieve the highest F1 score for MES 0 and 3 but performed consistently well across all classes, resulting in the highest overall macro-average F1 score.

Accuracy metrics followed similar patterns, with DenseNet201 and EfficientNetB3 achieving the highest accuracy for MES 0 (0.877 and 0.880 respectively), VGG19 for MES 1 (0.839), VGG19 for MES 2 (0.929), and DenseNet169 for MES 3 (0.968). These results indicate that different architectures exhibited specific strengths in identifying particular MES score categories.

### 3.2. Statistical Analysis

The results of the McNemar statistical analysis, which compare all models against VGG19 (the selected benchmark model based on the macro-averaged F1 score), are shown in Table 6. Statistically significant differences (p<0.05) were observed for several models. In particular, MobileNetV2, VGG16, ResNet34, EfficientNetB0, and EfficientNetB1 showed statistically significant differences from VGG19 in MES 0 performance. For MES 1, significant differences were observed with ViT and ResNet34. In MES 3, DenseNet121 and EfficientNetB4 demonstrated statistically significant performance differences compared to VGG19.

These findings reveal that despite the overall small numerical differences in overall performance metrics, certain architectures may be particularly well-suited for detecting specific MES score categories. This could provide important context for model selection, particularly where accurate identification of specific disease severity levels is critical.

## 4. Discussion

### 4.1. Model Architecture and Performance Analysis

This study adopts a comparative approach, evaluating state-of-the-art deep neural network classifiers for automated UC severity classification using endoscopic images. While the VGG19 network was found to perform the best based on the macro-averaged F1 score, the performance differences among the top-performing models were very small, with less than 0.01 separation between the five highest-scoring architectures. This suggests that multiple models would be viable candidates for deployment depending on specific requirements such as inference speed, memory constraints, or deployment environment. For example, smaller and faster models are suitable for deployment in settings with limited computing power, and where real-time analysis requirements are necessary.

The EfficientNet family demonstrated consistently strong results across its various configurations. Interestingly, EfficientNetB1 outperformed all its larger variants, EfficientNetB2-7, in terms of the macro-averaged F1 score, despite having significantly fewer parameters (6.5 M versus 63.8 M for EfficientNetB7). Similarly, MobileNetV2 exhibited competitive performance with only 2.2 million parameters, making it substantially more efficient than most other tested architectures. These findings challenge the conventional assumption that model size and complexity necessarily correlate with improved classification performance and are consistent with previous work on different domains [40].

The class imbalance inherent in the dataset used, despite being addressed through class weighting during training, noticeably affected model performance across different metrics. All models exhibited misleadingly high accuracy scores for the underrepresented classes (MES 2 and 3), primarily due to the dominance of true negatives in the evaluation. This imbalance paradoxically resulted in higher accuracy metrics for classes with fewer samples compared to the more abundant classes (MES 0 and 1). This observation reinforces the decision to prioritize the F1 score as the most reliable performance indicator, as it more effectively represents a model’s actual classification capabilities for minority classes by balancing precision and recall.

### 4.2. Comparison with Existing Methodologies

To directly compare the best-performing model in this study, VGG19, against other methodologies utilizing the LIMUC dataset, the bootstrapping resampling technique was employed and the network was re-evaluated several times to compute the weighted kappa score. Table 7 presents the quadratic weighted kappa (QWK) of VGG19, as well as the QWK scores reported in other studies.

Polat et al. [25] introduced a regression-based methodology. They treated the MES categories as independent classes rather than recognizing their ordinal relationship, by producing a single continuous value representing disease severity. The effectiveness of this methodology was demonstrated through testing across multiple CNN architectures. The most performant model was DenseNet121, achieving a QWK score of 0.854 (95% CI: 0.842–0.867) for the Mayo sub-score classification. This study’s VGG19 achieved 0.876 (95% CI 0.861–0.892), demonstrating slightly higher performance. The VGG19 model also showed stronger overall performance in class-specific metrics, achieving a mean F1 score of 0.753 with individual scores of 0.885, 0.702, 0.668, and 0.756 for MES 0–3 respectively, compared to DenseNet121’s reported macro F1 of 0.697. However, a detailed class-specific comparison was not possible as DenseNet121’s individual class F1 scores were not reported.

Pyatha et al. [41] proposed a self-supervised learning (SSL) methodology. The models ResNet50, ViT, and SwinB were combined with the MoCo-v3 (momentum contrast) self-supervised learning framework. The authors first pre-trained their model using self-supervised learning with the MoCo-v3 framework and one of the models as the backbone. After this pre-training phase, they fine-tuned the model for the specific task of UC grading using all available labeled training data. The best-performing model was MoCo-v3-SB with SwinB as the backbone with a mean F1 score of 0.711 and QWK score of 0.844 when fine-tuned using 100% of the samples. This research’s VGG19 model achieved higher mean F1 and QWK scores. Both approaches demonstrated particular strength in identifying both inactive and active disease states, with VGG19 having higher accuracy for MESs 1, 2, and 3. Both models showed similar patterns of class imbalance, with the strongest performance in detecting MES 0 (inactive disease), which is clinically valuable for monitoring disease remission. Individual class F1 scores for the MoCo-v3-SB model with SwinB as its backbone were not reported.

In another study by Polat et al. [42], the class distance weighted cross-entropy (CDW-CE) loss function was introduced. It was designed specifically for ordinal classification tasks. The function penalizes predictions more severely when they deviate further from the true class, with the *a* parameter determining how harshly such deviations are penalized. Their experiments revealed that the Inception-v3 architecture achieved the highest performance, with a quadratic weighted kappa (QWK) score of 0.872 when using CDW-CE with a margin, compared to 0.868 without a margin. These results were further validated across other architectures, with ResNet18 showing improvement from 0.857 to 0.860 and MobileNet-v3-L improving from 0.859 to 0.862. While the VGG19 architecture achieved a lower QWK score using standard cross-entropy loss compared to both variants of CDW-CE, this presents an opportunity for future research to investigate potential performance improvements by implementing CDW-CE loss with the VGG19 architecture.

### 4.3. Limitations and Future Perspectives

While this research’s implementation of the deep learning models demonstrated strong performance in UC severity classification, several limitations should be acknowledged.

First, the approach of using traditional cross-entropy loss may not optimally leverage the ordinal nature of Mayo scores. Future work should explore the incorporation of ordinal-aware loss functions such as CDW-CE, which has shown promise in recent studies. This could potentially improve the model’s understanding of the progressive nature of disease severity. Furthermore, this study utilized default hyperparameters across all model training. Future work should explore automated hyperparameter optimization techniques, to identify optimal configurations that could improve model accuracy and generalization capabilities.

Second, despite implementing weighted cross-entropy to mitigate class disparity, the models exhibited lower performance for underrepresented classes. This suggests that while class-weighting helps, it does not fully address the challenges posed by data imbalance. This imbalance could be problematic in clinical settings where accurate differentiation between moderate cases is crucial. Future research should investigate techniques to improve balanced performance across all Mayo scores. Furthermore, expanding the dataset with additional images, particularly for underrepresented categories, would likely enhance classification performance and lead to more robust, generalizable models. Data augmentation strategies, synthetic data generation, or other techniques should be explored to create a more balanced dataset. A critical next step for validating and assessing the generalizability and robustness of the current findings is to evaluate the best-performing models, particularly VGG19, on external, unseen ulcerative colitis (UC) image datasets from different medical centers and patient populations.

Third, unlike some comparative studies, k-fold cross-validation was not employed, which could provide more robust performance estimates. Although the validation strategy was chosen to facilitate direct comparisons, k-fold cross-validation was avoided for statistical comparison purposes, as this approach can lead to elevated Type I error rates when comparing machine learning algorithms [43]. Future work should include more in-depth validation approaches to better assess model generalizability and achieve the best possible results for clinical implementation.

Lastly, while the current implementation achieves strong performance, investigation into model compression techniques could make it more suitable for deployment in live resource-constrained clinical settings, as results of this research indicate that larger models do not necessarily outperform smaller ones. This could include exploring quantization, pruning, or knowledge distillation approaches while maintaining classification accuracy.

## 5. Conclusions

This research demonstrates the potential of deep learning models for ulcerative colitis (UC) severity classification using endoscopic imaging. Notably, multiple deep learning architectures showed robust performance in automatically assessing UC severity, with several models achieving consistently high classification accuracy. While VGG19 achieved the highest macro-averaged F1 score of 75.3% (with class-specific scores of 0.885, 0.702, 0.668, and 0.756 for MES 0-3) the strong performance across diverse model architectures with minimal statistical differences suggests that model selection should be guided primarily by deployment requirements rather than marginal performance gains. Notably, smaller models achieved competitive results despite having significantly fewer parameters, challenging conventional assumptions about model complexity and classification performance.

The consistency of results across different neural network models underscores the viability of AI-assisted UC severity assessment. To translate these promising research findings into clinical practice, future studies should focus on clinical validation. This would involve testing these automated classification systems in real-world clinical settings to support clinical decision-making.

By providing more consistent, and objective Mayo Endoscope Score assessments, these automated approaches are a significant step toward reducing the inter-observer variability that has traditionally complicated the determination of UC severity in clinical practice.

## Figures and Tables

**Table 1:** Model architecture parameters.

Model	Parameters
VGG16	28.1 M
VGG19	33.4 M
ViT-B/16	85.8 M
ResNet18	11.2 M
ResNet34	21.3 M
ResNet50	23.5 M
MobileNetV2	2.2 M
DenseNet121	7.0 M
DenseNet169	12.5 M
DenseNet201	18.1 M
EfficientNetB0	4.0 M
EfficientNetB1	6.5 M
EfficientNetB2	7.7 M
EfficientNetB3	10.7 M
EfficientNetB4	17.6 M
EfficientNetB5	28.3 M
EfficientNetB6	40.7 M
EfficientNetB7	63.8 M

**Table 2:** Performance metrics for MES 0.

Model	F1 Score	Accuracy	Precision	Sensitivity
MobileNetV2	0.873	0.864	0.883	0.863
ViT	0.867	0.852	0.843	0.892
VGG16	0.869	0.859	0.876	0.861
VGG19	0.885	0.874	0.874	**0.896**
ResNet18	0.884	0.873	0.873	0.895
ResNet34	0.873	0.863	0.875	0.872
ResNet50	0.879	0.868	0.867	0.892
DenseNet121	0.879	0.870	0.882	0.876
DenseNet169	0.877	0.867	0.875	0.878
DenseNet201	**0.887**	0.877	0.887	0.886
EfficientNetB0	0.879	0.870	0.883	0.875
EfficientNetB1	0.875	0.868	**0.890**	0.861
EfficientNetB2	0.878	0.869	0.880	0.876
EfficientNetB3	**0.887**	**0.880**	0.895	0.881
EfficientNetB4	0.884	0.875	0.885	0.883
EfficientNetB5	0.881	0.872	0.886	0.875
EfficientNetB6	**0.887**	0.878	0.886	0.888
EfficientNetB7	0.874	0.863	0.869	0.880

Note: Bold values indicate the best performance for each evaluation metric.

**Table 3:** Performance Metrics for MES 1.

Model	F1 Score	Accuracy	Precision	Sensitivity
MobileNetV2	0.676	0.819	0.655	0.699
ViT	0.639	0.814	0.673	0.608
VGG16	0.671	0.816	0.650	0.693
VGG19	**0.702**	**0.839**	**0.703**	0.701
ResNet18	0.678	0.832	0.701	0.656
ResNet34	0.665	0.823	0.681	0.651
ResNet50	0.673	0.826	0.685	0.662
DenseNet121	0.669	0.824	0.679	0.658
DenseNet169	0.682	0.825	0.669	0.695
DenseNet201	0.669	0.836	0.695	0.697
EfficientNetB0	0.678	0.824	0.672	0.683
EfficientNetB1	0.685	0.825	0.666	**0.705**
EfficientNetB2	0.672	0.823	0.674	0.670
EfficientNetB3	0.692	0.834	0.692	0.691
EfficientNetB4	0.684	0.831	0.690	0.678
EfficientNetB5	0.695	0.835	0.693	0.697
EfficientNetB6	0.696	0.836	0.696	0.697
EfficientNetB7	0.671	0.822	0.669	0.674

Note: Bold values indicate the best performance for each evaluation metric.

**Table 4:** Performance metrics for MES 2.

Model	F1 Score	Accuracy	Precision	Sensitivity
MobileNetV2	0.667	0.925	0.668	0.665
ViT	0.627	0.916	0.621	0.633
VGG16	0.657	0.926	0.683	0.633
VGG19	**0.668**	**0.929**	**0.697**	0.642
ResNet18	0.628	0.916	0.624	0.633
ResNet34	0.644	0.913	0.598	**0.698**
ResNet50	0.601	0.916	0.639	0.567
DenseNet121	0.626	0.917	0.629	0.623
DenseNet169	0.635	0.923	0.675	0.600
DenseNet201	0.626	0.921	0.641	0.614
EfficientNetB0	0.632	0.918	0.637	0.628
EfficientNetB1	0.659	0.924	0.662	0.656
EfficientNetB2	0.624	0.915	0.619	0.628
EfficientNetB3	0.631	0.917	0.630	0.633
EfficientNetB4	0.618	0.915	0.623	0.614
EfficientNetB5	0.616	0.916	0.628	0.604
EfficientNetB6	0.648	0.920	0.641	0.656
EfficientNetB7	0.640	0.921	0.657	0.623

Note: Bold values indicate the best performance for each evaluation metric.

**Table 5:** Performance metrics for MES 3.

Model	F1 Score	Accuracy	Precision	Sensitivity
MobileNetV2	0.774	0.966	**0.804**	0.745
ViT	0.732	0.960	0.770	0.698
VGG16	0.785	0.967	0.785	0.785
VGG19	0.756	0.963	0.775	0.738
ResNet18	0.722	0.956	0.712	0.732
ResNet34	0.747	0.962	0.771	0.725
ResNet50	0.777	0.964	0.750	0.805
DenseNet121	0.786	0.964	0.730	**0.852**
DenseNet169	**0.792**	**0.968**	0.792	0.792
DenseNet201	0.786	0.964	0.792	0.785
EfficientNetB0	0.768	0.964	0.758	0.779
EfficientNetB1	0.789	0.967	0.774	0.805
EfficientNetB2	0.748	0.960	0.739	0.758
EfficientNetB3	0.745	0.958	0.709	0.785
EfficientNetB4	0.782	0.965	0.748	0.819
EfficientNetB5	0.743	0.958	0.705	0.785
EfficientNetB6	0.766	0.965	0.787	0.745
EfficientNetB7	0.749	0.962	0.768	0.732

Note: Bold values indicate the best performance for each evaluation metric.

**Table 6:** *p*-Values for model comparisons against VGG19.

Model	MES 0	MES 1	MES 2	MES 3
MobileNetV2	**0.002**	1.000	0.620	1.000
ViT	0.777	**0.001**	0.897	0.430
DenseNet121	0.057	0.061	0.678	**0.001**
DenseNet169	0.117	0.859	0.298	0.134
DenseNet201	0.353	0.856	1.000	0.230
VGG16	**0.001**	0.757	0.890	0.248
ResNet18	1.000	0.052	0.897	1.000
ResNet34	**0.029**	**0.026**	0.126	0.856
ResNet50	0.771	0.095	0.056	0.064
EfficientNetB0	**0.041**	0.478	0.798	0.377
EfficientNetB1	**0.001**	0.931	0.807	0.099
EfficientNetB2	0.072	0.181	0.801	0.701
EfficientNetB3	0.172	0.729	0.902	0.265
EfficientNetB4	0.232	0.335	0.532	**0.043**
EfficientNetB5	0.064	0.927	0.389	0.265
EfficientNetB6	0.493	0.930	0.804	1.000
EfficientNetB7	0.174	0.247	0.734	1.000

Note: Bold values indicate statistical significance (p<0.05).

**Table 7:** Comparison of QWK scores between models.

Model	QWK Score
DenseNet121 (Polat et al.) [25]	0.854 (95% CI 0.842–0.867)
Swin-B (MoCo-v3) [41]	0.844
ResNet50 (MoCo-v3) [41]	0.669
ViT (MoCo-v3) [41]	0.750
Inception-v3 (CDW-CE) [42]	0.868
Inception-v3 (CDW-CE with margin) [42]	0.872
MobileNet-v3-L (CDW-CE) [42]	0.859
MobileNet-v3-L (CDW-CE with margin) [42]	0.862
ResNet18 (CDW-CE) [42]	0.857
ResNet18 (CDW-CE with margin) [42]	0.860
**VGG19 (Present Study)**	0.876 (95% CI 0.861–0.892)

## Data Availability

The original data presented in the study are openly available at https://zenodo.org/records/5827695#.ZF-92OzMJqs (accessed on 1 January 2025).
